# CD14 is a unique membrane marker of porcine spermatogonial stem cells, regulating their differentiation

**DOI:** 10.1038/s41598-019-46000-6

**Published:** 2019-07-10

**Authors:** Hyun-Jung Park, Won-Young Lee, Chankyu Park, Kwonho Hong, Hyuk Song

**Affiliations:** 1 Department of Stem Cell and Regenerative Technology, KIT, Konkuk University, 120 Neungdongro, Gwangjin-gu, Seoul, 05029 Republic of Korea; 2 Department of Beef Science, Korea National College of Agricultures and Fisheries, Jeonju-si, Jeonbuk, 54874 Republic of Korea

**Keywords:** Differentiation, Mammary stem cells

## Abstract

Molecular markers of spermatogonia are necessary for studies on spermatogonial stem cells (SSCs) and improving our understanding of molecular and cellular biology of spermatogenesis. Although studies of germ cell surface marker have been extensively conducted in the testes of rodents, these markers have not been well studied in domestic animals. We aimed to determine the expression pattern of cluster of differentiation 14 (CD14) in developing porcine testes and cultured porcine SSCs (pSSCs), as well as its role in pSSC colony formation. Interestingly, expression of CD14 was observed in porcine testes with PGP9.5-positive undifferentiated spermatogonia at all developmental stages. In addition, *in vitro* cultured pSSCs expressed CD14 and showed successful colony formation, as determined by fluorescence-activated cell sorting and flow cytometry. PKH26 dye-stained CD14-positive cells transplants were performed into the testes of recipient mice, which were depleted of both testicular germ and somatic cells from immunodeficiency mice and were shown to colonise the recipient testes. Moreover, a colony-forming assay showed that the development of pSSC colonies was disrupted by a high concentration of lipopolysaccharide. These studies indicated that CD14 is surface marker of early spermatogonia in developing porcine testes and in pSSCs, suggesting a role for CD14 in porcine spermatogenesis.

## Introduction

Spermatogonial stem cells (SSCs) are responsible for the producing of mature spermatozoa through a process of spermatogenesis^1^. The underlying mechanism of initiation of spermatogenesis by SSCs is an active area of research. Many specific markers of SSCs have been identified in mice and used for cell purification and to study SSC features *in vitro*^2,3^. Integrins alpha-6 and beta-1, glial cell-derived neurotrophic factor receptor alpha 1 (GFRα1), cluster of differentiation 9 (CD9), CD90, and cadherin 1 (CDH1) are specific surface markers of SSCs in mouse testes^2,4–6^. These markers are useful for SSC isolation and the production of transgenic mice^7^. However, the surface expression of known SSC markers has not been well established in domestic animals, thereby limiting SSC studies. Therefore, the description of unique cell surface markers is critical for the identification and isolation of SSCs from domestic animals. However, marker–function associations are less characterised in domestic animals than in mice^8^. A better knowledge of SSC markers in domestic animals is important to understand spermatogenesis and production of transgenic domestic animals.

CD14 is important components of the innate immune system^9^. It is abundant on the surface of myeloid cells^10^ but is not restricted to these cells in mice. In particular, strong induction of *CD14* mRNA expression in the testes, thymus, adipose, heart, uterus, spleen tissue, lung, liver and kidney of lipopolysaccharide (LPS)-treated mice were reported^11^. In addition, CD14 expression has been detected in a subpopulation of cryptorchidism testis cells enriched for SSCs^12^. The expression of *CD14* mRNA were also observed in the human and rat testes expressing Toll-like receptors (TLRs)^13,14^, although the role of CD14 in the testes is unclear. We have previously found that CD14 is expressed in porcine SSCs (pSSCs) using a next-generation sequencing approach; however, the role of CD14 in the testis have not been established^15^.

Therefore, the aim of this study was to determine the expression patterns of CD14 in developing porcine testes, cultured pSSCs, and testicular germ cells. The potential use of CD14 as a surface marker of germ cells in porcine and its putative functions are discussed.

## Results

### Localisation and expression of CD14 and PGP9.5 during porcine testis development

**Figure 1:**
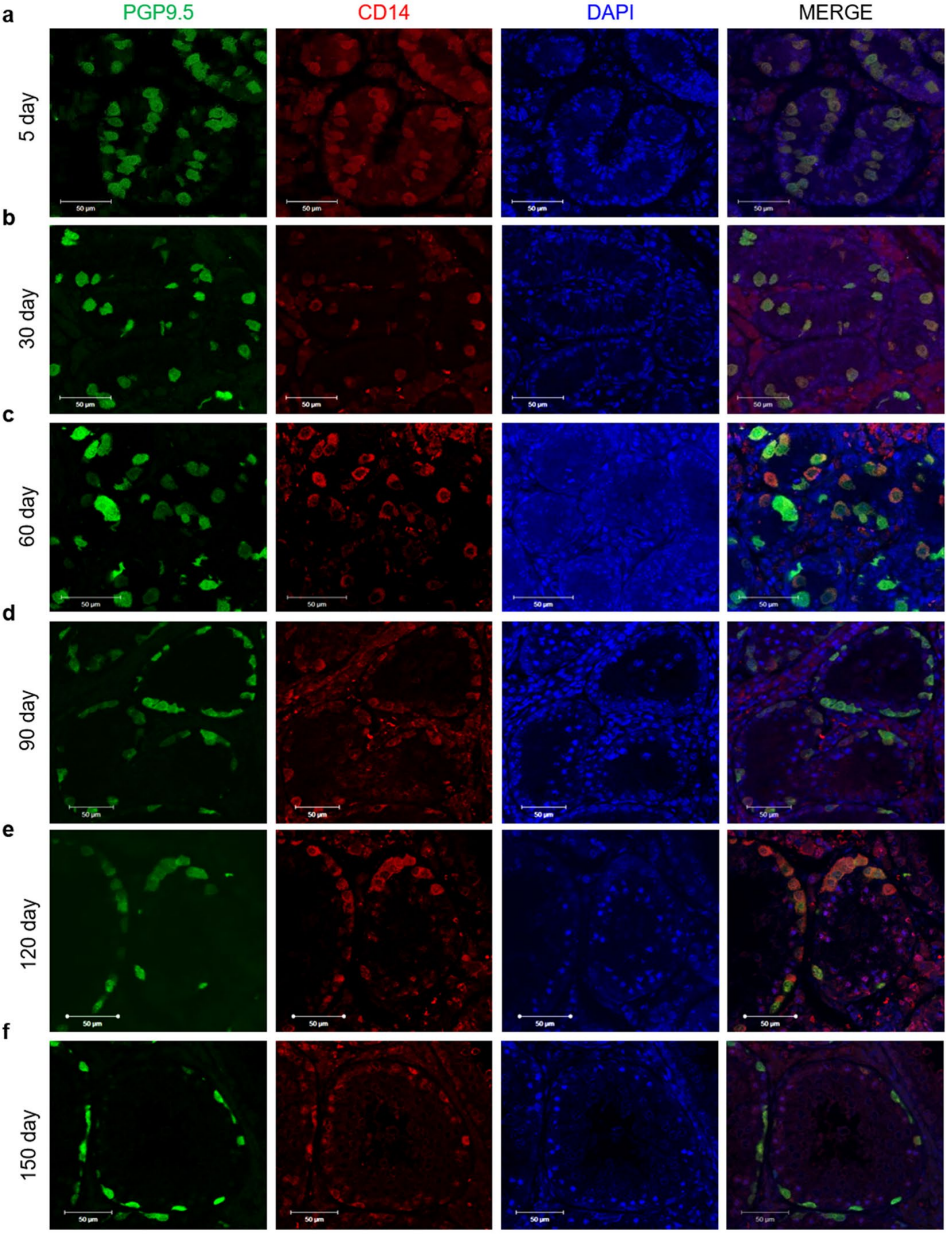
Localisation and expression of PGP 9.5 and CD14 at different developmental stages of porcine testes. Double immunolabelling of porcine testes was carried out using PGP9.5 and CD14 antibodies. CD14 (red) and PGP9.5 (green) expression was assessed in (**a**) 5-, (**b**) 30-, (**c**) 60-, (**d**) 90-, (**e**) 120-, and (**f**) 150-day-old porcine testes. Merged images show co-localisation of anti-CD14 and anti-PGP9.5 in testicular tissues and nuclei stained DAPI. Scale bars = 50 μm; n = 5, two pairs of testes.

We examined the localisation and expression of CD14 in the developing testis broad stage of porcine testes development which from postnatal day (p) 5 to p150 in porcine. The expression patterns of CD14 and PGP9.5, a specific marker for undifferenced spermatogonia in the porcine testis^16^, were compared at different stages by immunohistochemical analysis. Neonatal testes form 5-day-old piglets, PGP9.5-positive early spermatogonial cells were present in the centre of the seminiferous cord, and these cell in the luminal of seminiferous cord were translocated into basal compartment of seminiferous cords at p90. Interestingly, CD14-expressing cells were also located in the centre of the seminiferous cord, where PGP9.5-positive spermatogonial cells were found, in 5-, 30-, and 60-day-old testes (Fig. 1a–c) and were observed in PGP9.5-positive spermatogonia lining the basal lamina of seminiferous tubules in 90-, 120-, and 150-day-old testes (Fig. 1d–f).

### Comparison of CD14^+^ and PGP9.5^+^ cells from seminiferous tubules in pre-pubertal and post-pubertal porcine

**Figure 2:**
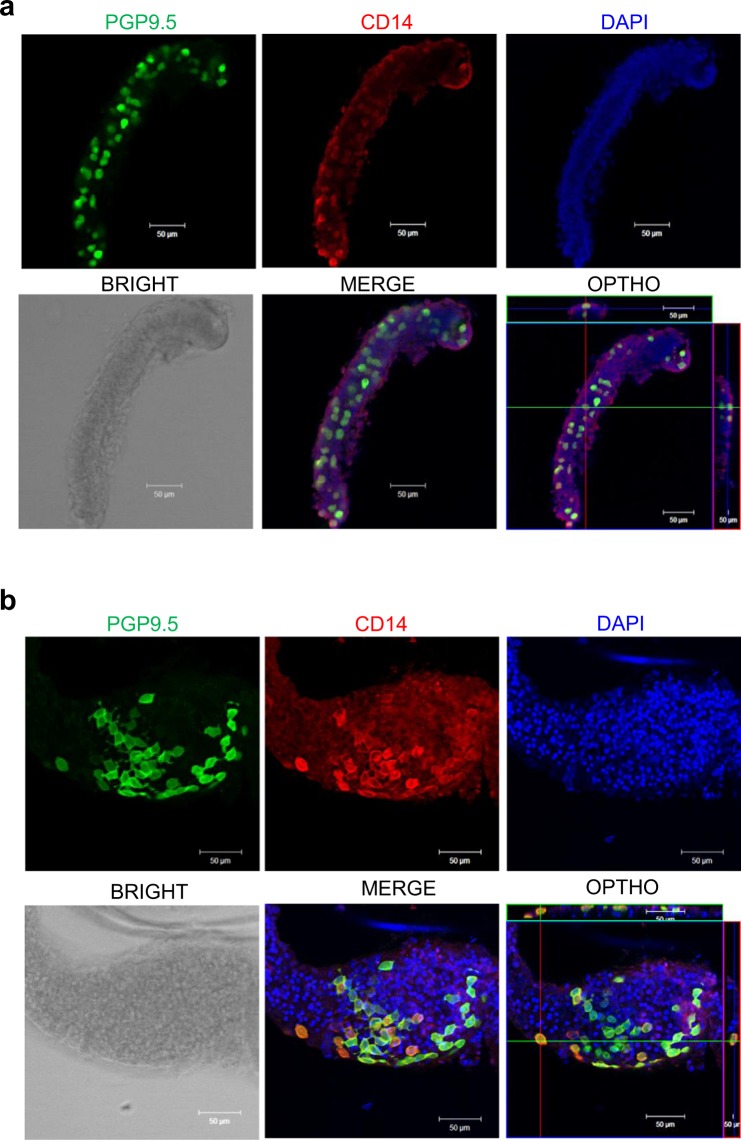
Immunohistochemistry of seminiferous tubules from 5- and 150-day-old porcine testes, double labelled with CD14 and PGP9.5 antibodies. Seminiferous tubules of (**a**) 5- and (**b**) 150-day-old porcine testes were used for whole-mount preparation. CD14^+^ fluorescence (red) was located at the same sites as PGP9.5^+^ fluorescence (green) in seminiferous tubules from both 5- and 150-day-old testes. Scale bars = 50 μm; n = 5, two pairs of testes.

Next, whole-mount immunostaining of CD14 and PGP9.5 of seminiferous tubules from 5- and 150-day-old porcine testes were carried out for confirming the CD14 and PGP9.5 co-expression. PGP9.5-positive undifferenced spermatogonia cells were detected in the basement membranes of seminiferous tubules, and coexpression of CD14 and PGP9.5 was detected in both testes from 5- and 150-day-old porcine (Fig. 2a,b). These founding were consistent with the previous immunostaining results for 5- and 150-day-old porcine testicular cells (Fig. 1a,f).

### Isolation of CD14^+^ cells from 5-day-old testes

**Figure 3:**
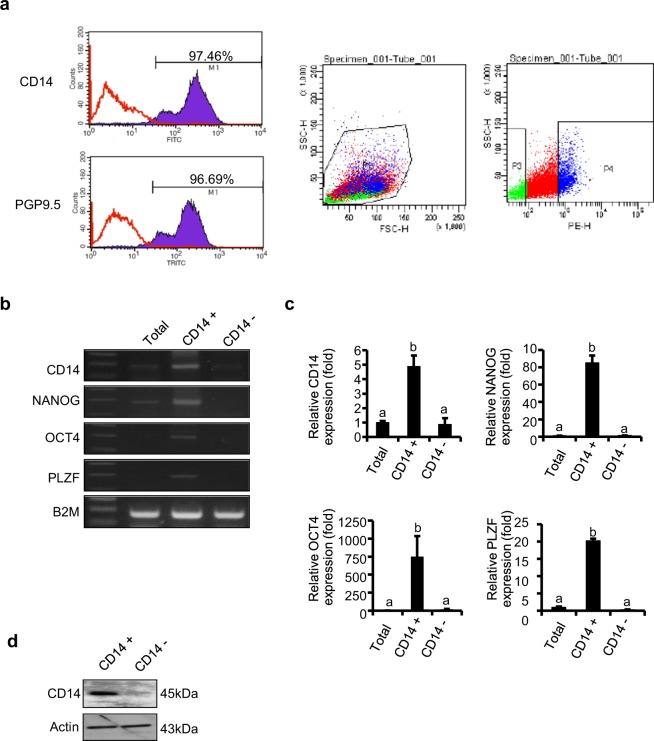
Flow cytometric and gene and protein expression analyses of CD14-expressing cells in porcine testicular cell populations. (**a**) Flow cytometric analysis of CD14 and PGP9.5 expression in porcine testicular cells. Most of the CD14^+^ cell population was PGP9.5-positive. (**b**,**c**) *CD14*, *NANOG*, *OCT4*, *PLZF*, and *B2M* gene expression in CD14^+^ and CD14^−^ cell populations from 5-day-old porcine testicular cells, determined by (**b**) RT-PCR and (**c**) qPCR. Relative mRNA levels are shown as the means ± SEM (n = 5). *B2M* was used as the endogenous control. (**d**) CD14 protein expression in CD14^+^ and CD14^−^ sorted cells, detected by immunoblotting.

To characterise CD14-positive cells, CD14-expressing cells were isolated using fluorescence-activated cell sorting (FACS) analysis. In total, 97.46% of the PGP9.5-positive cell population expressed CD14, and 96.69% of CD14-positive cells expressed PGP9.5 (Fig. 3a). In addition, CD14-positive cells were examined by real-time polymerase chain reaction (RT-PCR) and were shown to exhibit high levels of expression of stemness genes such as octamer-binding transcription factor 4 (*OCT4*), *NANOG*, and promyelocytic leukaemia zinc finger (*PLZF*) (Fig. 3b). The accuracy of the CD14 sorting results was verified by quantitative PCR (qPCR) and western blotting analysis (Fig. 3c–d). *CD14*, *NANOG*, *OCT4*, and *PLZF* were strongly expressed in CD14^+^ cells but not in CD14^−^ cells (Fig. 3c).

### CD14 expression in *in vitro* cultured pSSCs

**Figure 4:**
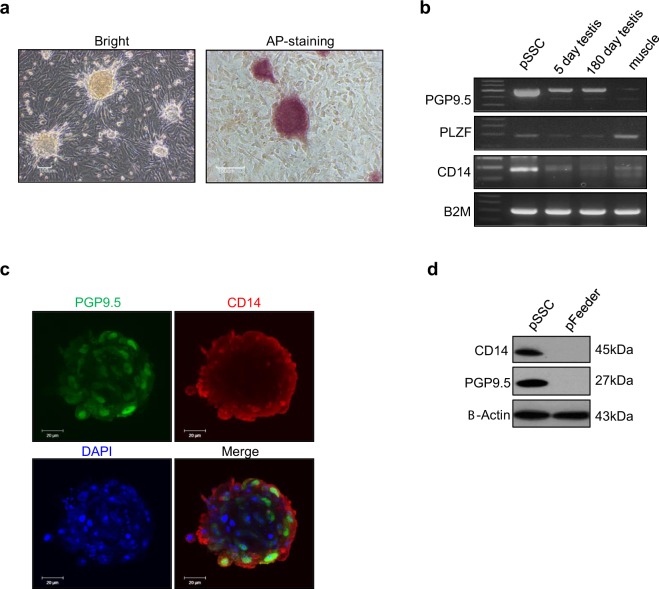
Characterisation of pSSC colonies. (**a**) The activity of Alkaline phosphatase (ALP) in SSC colonies (passage 5) from *in vitro* cultured cells derived from 5-day-old piglet testes. Scale bars = 100 μm. (**b**) The relative expression of *PGP9*.5, *PLZF*, *CD14*, and *B2M* mRNA in pSSCs were determined by RT-PCR. 5- and 150-day-old porcine testes, and porcine muscle tissues, used as a control. *B2M* was used as the endogenous control. (**c**) Immunocytochemistry of pSSC colonies using PGP9.5 (green), CD14 (red) antibodies and DAPI (blue). Scale bars = 20 μm. (**d**) Immunoblot analysis of CD14, PGP9.5, and β-actin in pSSC and pFeeder cell lysates; β-actin antibodiy was used as a loading control. The clear separation of pSSCs and pFeeder cells is supported by immunodetection of PGP9.5, a known pSSC marker.

CD14 protein expression, which was observed in all spermatogonia at various developmental stage of the porcine testis, was further evaluated in cultured pSSCs, successfully isolated from a 5-day-old porcine testis and showing alkaline phosphatase (ALP) activity (Fig. 4a). We examined the expression of PGP9.5, PLZF, and CD14 in pSSCs, 5- and 150-day-old whole porcine testes, and the porcine muscle as a negative control. *PGP9*.5 mRNA was stronger expressed in pSSCs than in the other types of samples. Both *CD14* and *PLZF* expression was clearly observed in pSSCs (Fig. 4b). To verify the expression of the CD14 protein in pSSC colonies, immunocytochemistry and confocal microscopy were used. Robust co-expression of PGP9.5 and CD14 in pSSC colonies were observed (Fig. 4c). Western blot analysis was performed to detect CD14 and PGP9.5 expression in both of pSSCs and testicular fibroblasts (pFeeder cells). PGP9.5, as a porcine spermatogonia marker, was only expressed by the pSSC population. Large amount of PGP9.5 and CD14 proteins exist exclusively in the pSSC lysate samples, but not detected in pFeeder cell lysate (Fig. 4d).

### Transplantation of CD14-positive porcine cells into the rodent testis. To prove

experimentally that certainly whether CD14^+^ cells were a spermatogonia in porcine testes, pSSCs obtained from a 5-day-old porcine testis and the cells were cultured for a month.

Red fluorescent PKH26-labelled pSSC colonies were formed after a week of culture.

**Figure 5:**
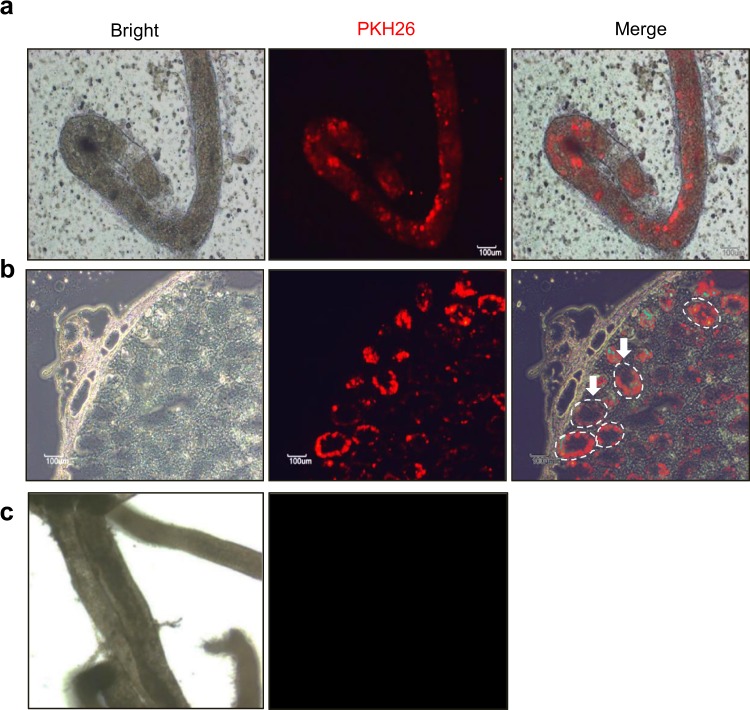
Results of transplantation of red fluorescent dye (PKH26)-labelled CD14^+^ porcine spermatogonia cells into the seminiferous tubules of busulfan-treated recipients (**a**) Fluorescent, brightfield, and merged images of seminiferous tubules from a 5-day-old porcine testis, with red fluorescent-labelled CD14^+^ cells. (**b**) Frozen sections of testes showing transplanted PKH26-labelled CD14^+^ cells (red) in the basement membranes of testes (white circle and arrow). (**c**) Brightfield and fluorescent images of mouse testis as negative control, which was non-transplanted. Scale bars = 100 μm.

PHK26-stained CD14^+^ cells from the pSSC colonies were transplanted into seminiferous tubules of recipient mice, which were depleted of both somatic and testicular germ cells due to busulfan treatment from immunodeficient models^17^ for the germ cell transplantation experiment. Eight weeks after xenogeneic transplantation, the testis of the recipients was collected Red fluorescent cells (CD14^+^) were clearly localized in basement membranes (Fig. 5a). In addition, analysis of frozen sections of testes reveal that the transplanted PKH26-labelled CD14^+^ cells properly localized and settled on the basal membranes of the seminiferous cord, where spermatogonia were found (Fig. 5b, white arrow), whereas the samples, seminiferous tubules of non-CD14^+^ cell-injected mice as negative control, showed no labelling (Fig. 5c).

### Effect of CD14 signalling in pSSCs *in vitro*

**Figure 6:**
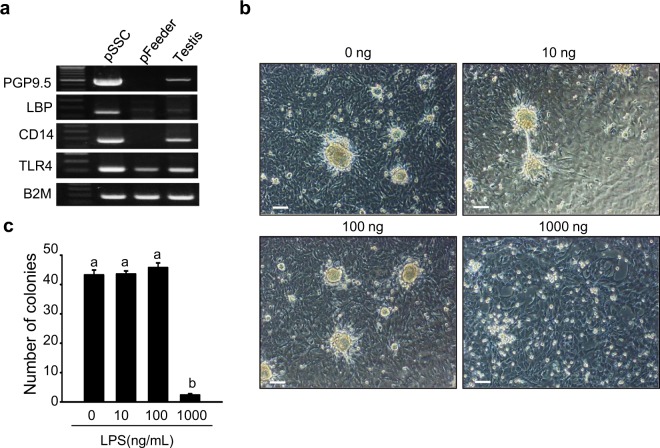
Effect of LPS treatment on *in vitro* pSSC culture. (**a**) *PGP9*.5, *LBP*, *CD14*, *TLR4*, and *B2M* gene expression in pSSCs, pFeeder cells, and 150-day-old whole testes, determined by RT-PCR. (**b**) Morphology of *in vitro* cultured pSSCs treated with various concentrations of LPS (0–1,000 ng/mL) for 48 h. Scale bars = 100 μm. (**c**) pSSC colony counts after 48 h of culture in media containing LPS (0, 10, 100, and 1,000 ng/mL). Data are the means ± standard deviation from five independent experiments.

CD14 signalling was evaluated in LPS-treated pSSCs *in vitro*. First, *PGP9*.5, LPS-binding protein (*LBP*), *CD14*, *TLR4*, and beta-2-microglobulin (*B2M*) were detected in pSSCs, pFeeder cells, and 150-day-old whole testis tissues (note that commercially available LBP antibodies were unsuitable for immunohistochemistry). The interaction of CD14, LBP and TLR4 has important role function as LPS signal transducers, leading to the activation of many molecules^18,19^. PGP9.5, LBP, CD14, and TLR4 expression was clearly detected in pSSCs, and the expression of TLR4 was also observed in pFeeder cells. Evaluation of the effects of various concentrations of LPS (0, 10, 100, and 1,000 ng/mL) on pSSC colony formation showed that a high concentration of LPS (1,000 ng/mL) induced the disruption of pSSC colonisation (Fig. 6b,c).

## Discussion

Some of research groups have identified specific biomarkers of pSSCs, such as PGP9.5^17^, PLZF, NANOG^20^, and stage-specific embryonic antigen-1 (SSEA-1)^21^. Recently, our group has detected Sal-like protein 4 (Sall4) and DEAD box protein 4 (DDX4) in spermatocytes and undifferentiated spermatogonia in porcine testes^22,23^. However, cell surface markers of undifferentiated porcine spermatogonia or pSSCs are not well defined. In porcine testes, several studies have reported CD90 (also known as THY1) as a surface marker of gonocytes^24^, phospholipase D (PLD) as a surface marker of undifferentiated spermatogonia in testis of prepubertal^25^, SSEA-1 as a suitable marker for porcine SSCs, including undifferentiated spermatogonia, in prepubertal boars^21^.

Previously, we have identified putative cell surface biomarkers of undifferentiated porcine spermatogonia, including chemokine receptor 1 (CCR1), CD14, and CD209, using next-generation sequencing technologies^15^. In mice, flow cytometric analysis confirmed that CCR2 and CD14 were expressed in a subpopulation of cryptorchid testicular cells enriched for SSCs^12^. However, the molecules of these cells may be markers of progenitor spermatogonia but not SSCs, as evidenced by the failure of CD14 and CCR2 fractions to produce spermatogenesis upon transplantation to recipient testes. In another study, expression of CD14 was reduced in different stage of germ cell such as preleptotene spermatocytes, pachytene spermatocytes, type B spermatogonia, and round and elongated spermatids in mouse testes^26^. Our results suggested that, unlike that in mouse testes, porcine CD14 is a putative cell surface biomarker of pSSCs or undifferentiated spermatogonia. Additionally, we examined the function of CD14 in male germ cells for the first time.

CD14 is a surface protein expressed on monocytes and macrophages^10^. CD34-positive haematopoietic stem cells derived from CD14-positive myeloid progenitors exhibit further enrichment and differentiate into macrophages^27^. In human, one study described that the subpopulation of CD14-positive cells from peripheral blood could be differentiated into numerous mesenchymal tissues, including cartilage, skeletal muscle, bone and fat tissues^28^. In addition, cells selected for positive CD14 expression are statistically more likely to form mesenchymal progenitor cell colonies than are both unsorted and negatively selected cells^29^. Furthermore, CD14 has been detected in mammary progenitor and cancer cells but displayed higher expression levels in a non-tumourigenic cell line^30^. These results indicate that CD14 is expressed in stem or progenitor cells, in which differentiation is regulated by LBPs. Recently, it has been reported that mRNA levels of *TLR2*, *TLR4*, and *CD14* are upregulated by LPS in porcine alveolar macrophages^31^. In addition, TLR4 have the ability to cooperate with CD14 on the host cell membrane to sense LPS in bacterial infections^32^. The roles of CD14 and LBP as bacterial pattern recognition receptors and in the modulation of the immune response by LPS are critical in the case of bacterial infection^33^. Although pSSC types differ among types of immune cells, our results showed clear expression of LBP, CD14, and TLR4 in pSSCs. Additionally, a high concentration of LPS (1,000 ng/mL) prevented pSSC colony formation *in vitro*. Clonogenic activity of murine SSCs is highly enhanced in an undifferentiated spermatogonial fraction^34,35^. Moreover, *in vitro* SSC culture conditions maintain clonogenic activity, resulting in a culture morphology similar to that of our pSSCs, and the resultant cells have a number of characteristics of undifferentiated spermatogonia found in mouse testes^36,37^. These studies suggest that the clonogenic activity of pSSCs is arrested by LPS signalling *in vitro* and indicate that LPS treatment decreases the stemness population in cultured pSSCs.

These data provide the first insight into the role of CD14 in pSSCs *in vitro* although further studies are needed to clarify the function of CD14 in pSSCs and porcine testes.

In conclusion, expression of CD14 was characterised in porcine testes with PGP9.5-positive undifferentiated spermatogonia. In addition, CD14 was detected in pSSCs cultured *in vitro*, and successful colony formation was observed by FACS. Furthermore, pSSC colony formation was disrupted by LPS treatment. These results suggest that CD14 is a suitable marker for early spermatogonia in developing porcine testes and in *in vitro* cultured pSSCs and probably play a role in porcine spermatogenesis.

## Methods

### Animals and sample preparation

Samples of testes (5- and 150-day-old porcine) were obtained from the National Institute of Animal Science (NIAS) and Sam Woo farm in South Korea. For each stage, including 5- and 150-day-old testes, three samples were used in this study. All procedures of this study were approved by the institutional Animal Care and Use Committee at NIAS (approval No. NIAS2015-120). Immunodeficient mice are being used as recipients for pSSC cell transplantation after obtaining approval from Konkuk University (approval No. KU17012).

### Immunohistochemistry and immunocytochemistry

As described previous studies^22^, approximately 1-cm^3^ pieces of porcine testes (5- and 150-day-old) were rinsed in phosphate-buffered saline (PBS) and tissue fixed in Bouin’s solution (Sigma–Aldrich, St. Louis, MO, USA) overnight at 4 °C. This samples were dehydrated via 120-min incubations with a 25–100% (v/v) ethanol gradient on a rotary shaker, then followed by paraffin embedding, sliced at 5 µm-thick sections using a rotary microtome (Thermo, Barrington, IL, USA), and samples were placed onto glass slides. Tissue samples were deparaffinized in xylene, rehydrated (100% to 50% ethanol), and equilibrated in water. Antigen retrieval was carried out by boiling section in 10 mM citrate buffer (pH 6.0) for 10 min. Nonspecific binding of antibodies were blocked by incubation the section for 30 min with blocking buffer (2% bovine serum albumin (BSA) with 0.05% Triton X-100 in PBS) at 22 °C. Samples were incubated overnight at 4 °C with diluted primary antibodies. The list of primary antibodies as follows:

CD14 (1:50 dilution; AbFrontier, Seoul, Korea)^15^ and PGP9.5 (1:500 dilution, 7863-0504; Serotec, Oxford, UK), followed by incubation with Alexa Fluor® 568-conjugated donkey anti-rabbit IgG and Alexa Fluor® 488-conjugated goat anti-mouse IgG (both Life Technologies, Carlsbad, CA, USA). To identify nuclei, samples were incubated with 1 µg/mL of 4′,6-diamidino-2-phenylindol (DAPI; Sigma–Aldrich) for 10 min and washed with PBS twice. Finally, coverslips were applied with mounting solution (Dako, Carpinteria, CA, USA).

For immunocytochemistry, three passages of *in vitro* cultured pSSC colonies were washed three times with PBS and fixed in 4% paraformaldehyde for 10 min. Membrane permeabilisation was then performed with PBS containing 0.05% Triton X-100 at room temperature for 10 min.

Nonspecific binding of protein was blocked with blocking solution (2% BSA in PBS) for 30 min at room temperature. Samples were incubated overnight at 4 °C with the CD14 (1:50 dilution) and PGP9.5 (1:500 dilution) primary antibodies, then washed with PBS and incubated for 1 h at room temperature with Alexa Fluor 568-conjugated donkey anti-rabbit IgG or Alexa Fluor 488-conjugated goat anti-mouse IgG. DAPI was added at a concentration of 1 µg/mL for 10 min for nuclear staining and mounted with mounting solution (Dako).

Fluorescence microscopy images of both samples, pSSC colonies and tissue, were collected using a confocal microscope (LSM 700; Carl Zeiss, Oberkochen, Germany).

### pSSC isolation, *in vitro* culture, and colony formation

The pSSC isolation and *in vitro* culture were performed as described previously^15^. Briefly, testis samples were collected from 5-day-old crossbred piglets (Sam Woo breeding farm), the tunica albuginea were removed, digested with an enzyme mixture solution (DNase I and collagenase IV in PBS) at 37 °C for 15 min, and filtration through a 40-µm nylon mesh. Red blood cells (RBCs) were removed from RBC lysis buffer (Sigma-Aldrich) which eliminate erythrocytes. Then isolated cells were seeded in 0.2% (w/v) gelatine-coated plates at a density of plates (2 × 10^5^ cells/well on of 12 well plate) and then, cultured in StemPro-34 medium (Gibco) at 31 °C and 5% CO_2_. Upon reaching 80% confluence, pSSCs, on testicular fibroblasts (pFeeder cells), were trypsinised using 0.005% trypsin (Gibco) every 6–7 days. Newly prepared pFeeder cells were seed into 0.2% (w/v) gelatine-coated plates and seed the pSSC (0.5 × 10^5^ cells) onto the pFeeder cells. Typically, at least two passages were required for colony formation. Next, colony formation assay carried out with LPS. pSSCs at the third passage were seeded on gelatine-coated 12 well plates (2 × 10^5^ cells/well) and cell were treated with various dose of LPS (0, 10, 100, and 1,000 ng/mL) and fresh media for 48 h. Images of colony formation by LPS-treated pSSCs were collected using a microscope (Nikon, Tokyo, Japan) after 48 h of incubation with LPS, and colonies were counted.

### RNA extraction, RT-PCR, and qPCR

Total RNA from testis tissues of 5- and 150-day-old porcine, pSSC colonies (passage 3), and the porcine muscle were extracted using the on-column DNase treatment (Qiagen) and RNeasy mini kit (Qiagen, Hilden, Germany). Complementary DNA (cDNA) was synthesised from 1 µg of total RNA using an RT-PCR premix kit (iNtRON, Seongnam, South Korea).PCR amplification of genes was achieved using following method: 35 cycles at 95 °C for 30 s, 57 °C for 20 s, and 72 °C for 20 s. Primers were designed using Primer3 software (http://frodo.wi.mit.edu) and primer sequences are listed in Table 1. qPCR was carried out with a total volume of 20 μL, containing 10 ng of cDNA samples and 1 pM each primer in the iQ SYBR Green supermix reaction buffer (Bio-Rad Laboratories, Hercules, CA, USA). The PCR conditions included denaturation and polymerase activation step at 94 °C for 1 min and then 40 cycles consisting of 94 °C for 10 s, 57 °C for 10 s, and 72 °C for 20 s. The cycle threshold (Ct) values were normalised against the expression level of *B2M* gene.

**Table 1:** List of porcine-specific primers.

Gene	Primer sequence
*B2M*	F: TTCACACCGCTCCAGTAG R: CCAGATACATAGCAGTTCAG
*CD14*	F: ACCACCCTCAGACTCCGTAAT R: ATAGGTCCAGGGTGGTGAGAG
*NANOG*	F: CCTCCATGGATCTGCTTATTC R: CATCTGCTGGAGGCTGAGGT-3
*OCT4*	F: GTTCTCTTTGGGAAGGTGTT R: ACACGCGGACCACATCCTTC
*PLZF*	F: GGCTCGGTATCTCAAGAACATC R: ACTGCCCTATGGTCATCAAACT
*PGP9*.5	F: GAGATGCTGAACAAAGTGCTG R: CATGGTTCACCGGAAAAGG
*LBP*	F: AGCCGAATGGTCTACTTTGC R: AAGGAGTTGGTGGTCAGTCG
*TLR4*	F: GGCATCATCTTCATCGTCCT R: TCCTCCCACTCCAGGTAGGT

### Alkaline phosphatase staining

ALP activity was found in pSSCs using an ALP kit (Sigma–Aldrich) according to the manufacturer’s procedure. Briefly, cultured pSSCs (passage 3) and testicular feeder cells were washed twice with PBS, fixed with a citrate/acetone/formaldehyde solution for 30 s. The fixed cells were then washed with PBS, stained with an alkaline dye mixture (naphthol AS-BI alkaline and FBB alkaline), and incubated at 22 °C for 30 min. After incubation, the dye was removed, and the cells were rinsed twice in deionised water before collecting of imaging.

### Seminiferous tubules whole mount preparation

The whole-mount immunostaining has been described previously^38^. A whole mount immunohistochemical technique was used with the CD14 and PGP9.5 antibodies. Testes samples form five-day-old neonatal piglet were encapsulated and seminiferous tubules were dissociated with collagenase IV in PBS and fixed overnight at 4 °C in 4% paraformaldehyde. After samples washed in PBS for 1 h, samples were dehydrated in an methanol series of 25–100% in water for 10 min and permeabilization of cell in MeOH/dimethyl sulfoxide/H_2_O_2_ (4:1:1, v/v/v) were carried out for 3 h at room temperature. Next, samples were rehydrated in MeOH (50% and 30%) in PBS for 10 min. The samples were blocked with a blocking buffer containing 2% BSA and 0.5% Triton X-100 in PBS for 2 h and then incubated overnight at 4 °C with the PGP9.5 (1:200) and CD14 (1:50) antibodies in the blocking buffer. After washing in PBS, the samples were incubated overnight with secondary antibodies (Alexa Fluor 568 anti-mouse IgG and Alexa Fluor 488 anti-rabbit IgG), diluted 1:1,000 in blocking buffer at 4 °C. Samples were mounted with mounting reagent (Dako), and images were collected using a confocal microscope (LSM 700; Carl Zeiss).

### Cell sorting and FACS analysis

Porcine testicular cells were isolated using our routine cell culture techniques as previously described^15^. Briefly, testes from 5-day-old crossbred piglets were detunicated and digested with an enzyme mixture solution (collagenase IV and DNase I in PBS) at for 15 min at 37 °C and strained through a 40-µm nylon mesh to obtain single testicular cells for FACS. RBCs were eliminated by treatment with RBC lysis buffer (Sigma–Aldrich), and the remaining cells were washed with PBS. These single cells were incubated with anti-rabbit CD14 and anti-mouse PGP9.5 antibodies diluted in a diluent buffer (3% foetal bovine serum in PBS) for 60 min at room temperature. After washes, the cells were stained with secondary antibodies (Alexa Fluor 488 anti-rabbit IgG and Alexa Fluor 546 IgG, 1:1000 diluted in PBS) for 60 min at room temperature and then these cells were resuspended in PBS and analysed using a FACSAria apparatus (BD Biosciences, San Jose, CA, USA). *CD14*, *NANOG*, *OCT4*, *PLZF*, and *B2M* gene expression profiling of the sorted CD14^+^ cells were performed by qPCR and RT-PCR.

### Recipient mice and donor cell transplantation

The busulfan-treated recipient mouse model has been described in our previous studies^38,39^. Briefly, 10-week-old BALB/c immunodeficient mice (n = 4; Orient Bio, Inc., Seongnam, South Korea) were used as recipient animals because we chose these recipients to avoid immunological rejection of donor cells. The mice were injected with 40 mg/kg busulfan (Sigma–Aldrich) at least 5 weeks before CD14^+^cell transplantation to deplete germ cells in the testis of recipients^7^.

CD14^+^ cells membranes were labelled with 2 μM PKH26 red fluorescent membrane linker dye (Sigma–Aldrich) for 3 min after sorting, then washed five times with medium (Dulbecco’s Modified Eagles Medium) and resuspended in 10% foetal bovine serum/DMEM. An aliquot 10 μL (1 × 10^5^ cells) of L PKH26-labelled CD14^+^ cells was injected into each recipient testis. Eight weeks later, the recipient mice were sacrificed and testes were harvested for analysis. The image of localisation of PKH26-labelled CD14^+^ cells in the seminiferous tubules was collected by fluorescence microscopy (Nikon).

### Immunoblotting

Protein samples were prepared from pSSCs, whole porcine testes (5- and 150-day-old), and muscle tissues in RIPA buffer (Thermo Fisher Scientific) with a protease inhibitor cocktail (Roche). Samples containing equal quantities of protein were separated by electrophoresis in 4% to 20% Mini-PROTEAN® TGX (Bio-Rad) gels via SDS-PAGE, and then separated proteins were transferred to polyvinylidene difluoride membranes (PVDF).

After blocking with blocking solution (1% BSA in PBS) for 1 h 25 °C, and then the membranes were probed with specific primary antibodies at 4 °C overnight. The following primary antibodies were used: CD14 (1:500 dilution; AbFrontier) and β-actin (1:1,000 dilution; Santa Cruz Biotechnology). Membrane were washed with washing buffer (0.1% tween-20 in PBS) for 15 min each, the membranes incubated for 1 h with appropriate secondary antibodies such as anti-rabbit and anti-mouse IgG (1:1,000 dilutions; Santa Cruz Biotechnology). The blots were visualised using an enhanced chemiluminescence substrate (Pierce) and the HyBlot CL autoradiography film (Denville Scientific, Metuchen, NJ, USA). Full-length pictures of the blots presented in the Supplementary file. 

### Statistical analysis

All data were presented means ± standard error of the mean (SEM) and statistically analysis was performed using SPSS statistical package ver. 15.0 for Windows (IBM Corp., Somers, NY, USA). The significance differences between control and experimental samples were determined by one-way analysis of variance, followed by Tukey’s honest significant.

## Supplementary information

Supplementary information
