# Supplementary material for: CD14 is a unique membrane marker of porcine spermatogonial stem cells, regulating their differentiation
Source: Sci Rep. 2019 Jul 10;9:9980. doi: 10.1038/s41598-019-46000-6 (PMC6620343; doi:10.1038/s41598-019-46000-6)

CD14 is an unique membrane marker of porcine spermatogonial stem cells, regulating their differentiation

Hyun-Jung Park[1], Won-Young Lee[2], Chankyu Park[1], Kwonho Hong[1] and Hyuk Song[1*]

[1]Department of Stem Cell and Regenerative Technology, KIT, Konkuk University, 120 Neungdongro, Gwangjin-gu, Seoul 05029, Republic of Korea

[2]Department of Beef Science, Korea National College of Agricultures and Fisheries, Jeonju-si 54874, Republic of Korea

Running title: CD14 is a putative porcine spermatogonial stem cell marker

*Correspondence: Prof. Hyuk Song, Department of Stem Cell and Regenerative Technology, KIT, Konkuk University, 120 Neungdongro, Gwangjin-gu, Seoul 05029, Republic of Korea

Tel: +82-43-840-3522; e-mail: songh@konkuk.ac.kr; fax:+82-2-450-1044

Supplementary Information

Supplementary data of Figure3

CD14 and β actin protein expression in CD14+ and CD14- cell lysate was detected by western blot. Image are presented full-length gel with size marker (KDa)

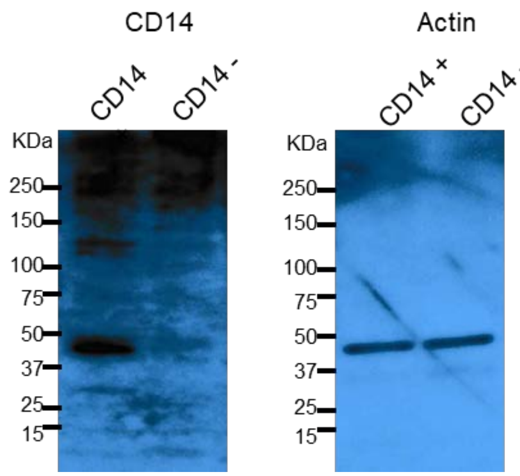

Supplementary data of Figure 4

CD14, PGP9.5 and β actin protein expression in pSSC and pFeeder cells lysate was detect by western blot. Image are presented full-length gel with size marker (KDa)

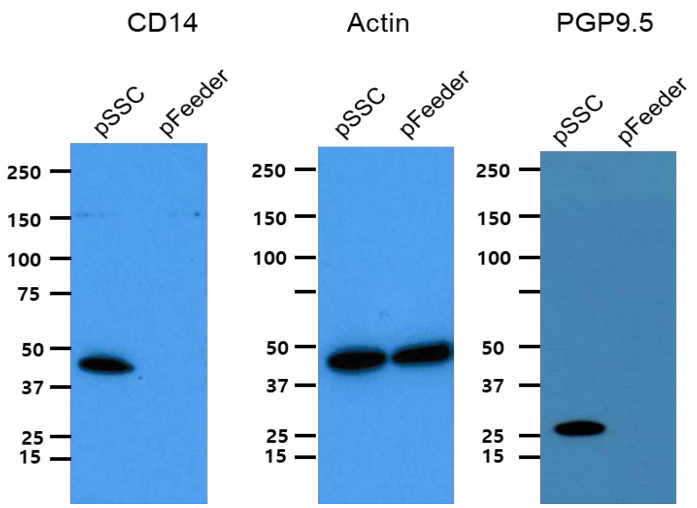

Supplement: Supplementary file 1 — Supplementary information [file 41598_2019_46000_MOESM1_ESM.pdf]
